# Occurrence of subdural hematomas in Dutch glutaric aciduria type 1 patients

**DOI:** 10.1007/s00431-016-2734-6

**Published:** 2016-05-31

**Authors:** Marloes E.M. Vester, Gepke Visser, Frits A. Wijburg, Francjan J. van Spronsen, Monique Williams, Rick R. van Rijn

**Affiliations:** Department of Radiology, Amsterdam Medical Center, Room G1-213, Meibergdreef 9, 1105 AZ Amsterdam, The Netherlands; Department of Forensic Medicine, Netherlands Forensic Institute, The Hague, The Netherlands; Department of Metabolic Diseases, Wilhelmina Children’s Hospital-University Medical Center Utrecht, Heidelberglaan 100, 3584 CX Utrecht, The Netherlands; Department of Pediatrics—Academic Medical Center, Meibergdreef 9, 1105 AZ Amsterdam, The Netherlands; Division of Metabolic Diseases, Beatrix Children’s Hospital-University Medical Center Groningen, University of Groningen, Hanzeplein 1, 9713 GZ Groningen, The Netherlands; Sophia Children’s Hospital-Erasmus Medical Center, ‘s-Gravendijkwal 230, 3015 CE Rotterdam, The Netherlands

**Keywords:** Metabolic disorder, Glutaric aciduria type 1, Subdural hematoma, Abusive head trauma, Forensic radiology

## Abstract

Patients with glutaric aciduria type 1 (GA1), a rare inherited metabolic disorder, have an increased risk for subdural hematomas (SDHs). GA1 is therefore generally included in the differential diagnosis of children presenting with SDHs. This retrospective cohort study reviews all 25 registered, in the Dutch Diagnosis Registration for Metabolic Disorders, GA1 patients in the Netherlands. This was done between May 2014 and November 2014 to determine the lifetime incidence of SDHs in this population. Seventeen patients were diagnosed either due to clinical symptoms or because of family members with GA1. One out of these 17 had a SDH. This patient showed widened Sylvian fissures on MRI, characteristic for GA1. Eight patients were diagnosed by newborn screening. Three of them had neuroimaging results, and none of them had SDHs. This study shows an overall lower incidence (4.0 %) of SDHs in patients with GA1 than reported in the literature (20–30 %).

*Conclusion*: This finding, in combination with the fact that SDHs in GA1 appear to occur only in the presence of characteristic brain abnormalities on imaging, we recommend that GA1 should not routinely be a part of the differential diagnosis of children with unexplained SDHs in the absence of imaging characteristics suggestive of GA1.

**Table:** 

**What is known**: • *Glutaric aciduria type 1 is a rare metabolic disorder predisposing children to subdural hematoma development due to brain abnormalities*. • *Because of these subdural hematomas*, *glutaric aciduria type 1 testing is part of abusive head trauma work*-*up*.
**What is new**: • *The overall subdural hematoma incidence in glutaric aciduria type 1 patients is much lower than previously reported and only occurs in case of predisposing brain abnormalities*.

## Introduction

Glutaric aciduria type 1 (GA1, OMIM#231670) is a rare, autosomal recessive inborn error of metabolism caused by a deficiency of glutaryl-CoA dehydrogenase (GCDH). This enzyme is involved in the breakdown of the amino acids lysine, hydroxylysine and tryptophan [13]. Deficiency of GCDH can result in the accumulation of intermediate breakdown products, particularly of glutaric acid and 3-hydroxy-glutaric acid which are neurotoxic [16]. The prevalence of GA1 is estimated to be 1 in 100,000 live newborns [18]. The main clinical presentation consists of macrocephaly and encephalopatic crisis, often triggered by a minor infection within the first 36 months of life. As a consequence of an encephalopatic crisis, other symptoms develop including orofacial dyskinesia, choreoathetosis, epilepsy, dystonia, and cognitive impairment [15]. Although up to 25 % of untreated patients remain completely asymptomatic, GA1 is associated with high morbidity and mortality [6]. Treatment consists of a low lysine diet with supplementation of essential amino acids and carnitine which may prevent the development of neurological damage in asymptomatic patients [14, 15]. Because of the importance of an early start of treatment, aiming at prevention of irreversible central nervous system (CNS) damage, GA1 is included in the newborn screening (NBS) panel in many countries [13], including the Netherlands where it was introduced in 2007.

Neuroradiological imaging may reveal several abnormalities in GA1 patients. The most characteristic anomaly is the “batwing sign” due to reduced operculization of the brain, also referred to as widening of the Sylvian fissures, which is reported in up to 93 % of patients [24, 26]. This can be accompanied by bilateral frontotemporal cortical atrophy and enlargement of ventricular spaces and mesencephalic cisterns caused by a delayed start of treatment or due to acute encephalopathic crises [1, 24]. In addition, subdural hematomas (SDHs) have been reported as complications in GA1 patients. Although brain abnormalities are generally detected on brain imaging performed during or after a metabolic crises, studies in patients detected by NBS show that extensive radiological changes may already be present very early in life in the absence of clinical disease [14, 23], demonstrating that at least part of the morphological changes originate during intra-uterine life [9]. Furthermore, in utero MRI studies have established a maturational delay of the brain leading to hypoplasia and immature gyral patterns [5]. These in utero abnormalities can normalize later on however [17, 19].

Usually SDHs in children with GA1 occur in cases with preexisting neuroimaging abnormalities [25]. The cortical atrophy and expanded cerebrospinal fluid (CSF) spaces may cause stretching of the cortical veins, which may cause them to rupture [9]. This mechanism explains why GA1 patients are considered to be more vulnerable to the development of subdural hematomas after minor trauma compared to healthy children. Conversely, cerebrovascular changes like arteriolar dilation and increased cerebral blood volume could be the cause of CSF expansion and SDHs by creating venous hypertension [22]. The incidence of SDHs in GA1 children has been estimated at 20–30 % outside the Netherlands [20]. In pediatric literature, GA1 is a routine part of the work-up of SDH in case of suspected abusive head trauma (AHT) [13].

Patients with SDHs associated with undiagnosed GA1 have been erroneously diagnosed as AHT [6], and as a consequence, urine, blood, and/or enzymatic GA1 investigations to exclude this disorder are advised in case of SDH in suspected AHT. However, a priori chance of an unknown case of GA1 when dealing with a SDH with an otherwise normal brain is very low, especially since the introduction of GA1 in NBS programs. The aim of this study therefore is to investigate the prevalence of SDHs in a Dutch cohort of patients with GA1 and to assess the related neuroimaging results.

## Materials and methods

A retrospective review of all Dutch GA1 patients was performed between May 2014 and November 2014. Dutch GA1 patients are registered in the Dutch Diagnosis Registration for Metabolic Disorders, founded in 2001 [2]. This is a collaborative database of the seven metabolic centers in the Netherlands and formed the basis of our study. In four of these centers, i.e., the Academic Medical Center Amsterdam, the Erasmus Medical Center in Rotterdam, the University Medical Center Groningen, and the University Medical Center Utrecht, patients with GA1 were seen for follow-up. All patients with confirmed laboratory GA1 diagnosis at the time of review were included, no exclusion criteria were used.

Patient records were screened by one researcher (MV) for patient demographics and clinical history. Neuroimaging results were evaluated, and in cases of the presence of a SDH, the radiological imaging was reevaluated by a forensic pediatric radiologist (RvR).

All extracted data were handled anonymously. The internal review board of the Academic Medical Center Amsterdam has issued a waiver for retrospective anonymized patient medical record studies, specific approval for this study therefore was not mandatory.

## Results

Twenty-five patients with GA1 were identified, 17 males and 8 females. All but one were alive at the time of the study (Table 1). Four patients (16.7 %) had GA1 relatives who were also included in this study. Patients 1 and 5 are brothers, as are patients 3 and 4. Patients were divided into two groups. The first group of patients, who were diagnosed before NBS for GA1 was introduced, consisted of 17 patients (70.8 %) with a median age at time of diagnosis of 2.5 years (range 0 months–8 years). The second group with eight patients was identified by NBS. All patients received appropriate therapy from the time of diagnosis (Table 2).

**Table 1:** Dutch glutaric aciduria type 1 population identified by clinical presentation

Patient	Center	Age^a^	Sex	Head circumference, standard deviation	CT or MRI	Lobe atrophy	Open opercula	Basal ganglia attenuation	Widened CSF spaces or ventricles	White matter abnormalities	Arachnoid cysts	Subdural hematoma
1	AMC	11	M	0 SD	No	NR	NR	NR	NR	NR	NR	NR
2	AMC	14	F	+1 SD	MRI	No	Yes	No	Yes	Yes	No	No
3	AMC	15	M	−1 SD	MRI	No	Yes	Yes	Yes	No	No	No
4	AMC	16	M		MRI	No	Yes	No	No	No	No	No
5	AMC	19	M	+2.5 SD	MRI + CT	Yes	Yes	No	Yes	Yes	No	No
6	AMC	21	M	+2 SD	No	NR	NR	NR	NR	NR	NR	NR
7	ErasmusMC	10	F	+2 SD	MRI	Yes	Yes	No	No	No	No	No
8	ErasmusMC	16	M	+2 SD	CT + MRI	Yes	Yes	Yes	No	Yes	Yes, bilateral	No
9	ErasmusMC	20	M	+2 SD	CT	No	Yes	No	Yes	No	No	No
10	ErasmusMC	22	M	+0.2 SD	CT	No	No	No	No	No	No	No
11	ErasmusMC	25	M	+2.5 SD	MRI	Yes	Yes	Yes	Yes	Yes	No	No
12	ErasmusMC	27	F	Unknown	MRI	No	No	No	No	No	No	No
13	ErasmusMC	33^b^	M	+1.5 SD	CT	Yes	Yes	No	Yes	No	No	No
14	UMCU	11	M	>3 SD	CT + MRI		Yes	Yes	Yes	Yes		Yes, bilateral
15	UMCU	12	M	0 SD	MRI	No	Yes	No	Yes	Yes	No	No
16	UMCG	10	M	−2 SD	MRI	No	Yes	Yes	No	No	No	No
17	UMCG	22	F	+2 SD	MRI	No	No	Yes	No	No	No	No

*AMC* Academic Medical Center Amsterdam, *ErasmusMC* Erasmus Medical Center Rotterdam, *UMCG* University Medical Center Groningen, *UMCU* University Medical Center Utrecht, *NR* Not reported

^a^Age in years at time of review in October 1, 2014 or age of dying

^b^Age at time of death

**Table 2:** Dutch glutaric aciduria type 1 population identified by newborn screening

Patient	Center	Age^a^	Sex	Head circumference, standard deviation	CT or MRI	Lobe atrophy	Open opercula	Basal ganglia attenuation	Widened CSF spaces or ventricles	White matter abnormalities	Arachnoid cysts	Subdural hematoma
18	AMC	3	F	+1 SD	MRI	No	Yes	Yes	No	No	No	No
19	AMC	4	M	−1 SD	No	NR	NR	NR	NR	NR	NR	NR
20	ErasmusMC	11 months	F	+1.8 SD	CT	No	Yes	Yes	Yes	No	No	No
21	ErasmusMC	3	F	+0.2 SD	No	NR	NR	NR	NR	NR	NR	NR
22	ErasmusMC	3	M	−1 SD	No	NR	NR	NR	NR	NR	NR	NR
23	ErasmusMC	5	F	0 SD	No	NR	NR	NR	NR	NR	NR	NR
24	UMCU	6	M	+2.5 SD	No	NR	NR	NR	NR	NR	NR	NR
25	UMCG	7	M	+2 SD	MRI	No	Yes	No	No	No	No	No

*AMC* Academic Medical Center Amsterdam, *ErasmusMC* Erasmus Medical Center Rotterdam, *UMCG* University Medical Center Groningen, *UMCU* University Medical Center Utrecht, *NR* Not reported

^a^Age in years at time of review in October 1, 2014

According to the Central Bureau of Statistics (CBS) of the Netherlands, there were approximately 6,310,000 children born between 1980 and 2014 and a 99.8 % compliance with the NBS program. Therefore, the incidence of GA1 in the Netherlands is approximately 1 in 200,000 persons.

## Clinical diagnosis group

**Fig. 1:**
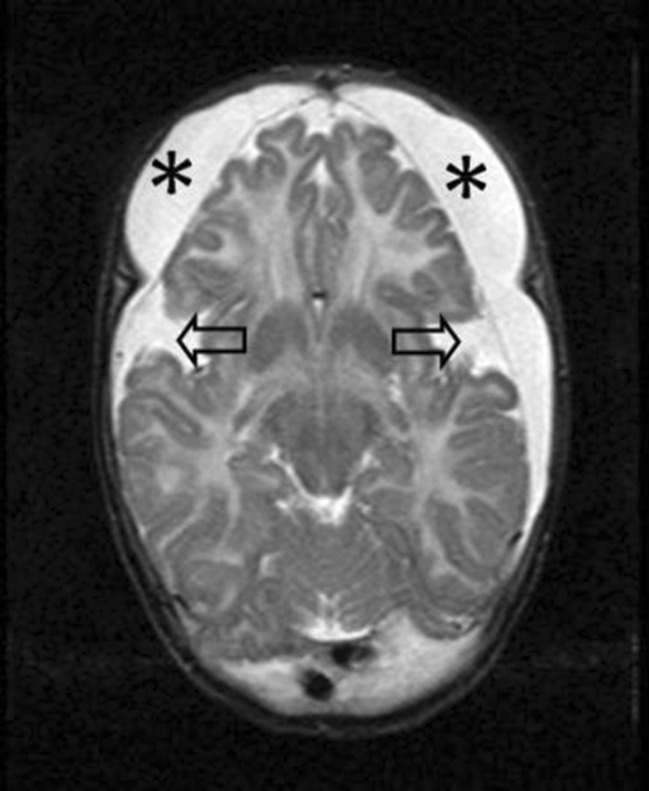
T2-weighted MRI of a boy at 6 months old (patient no. 14) with subdural collections. MRI settings: T2 SE, slice thickness 6 mm, TR: 3465, TE 150, flip angle 90°, NSA: 1. Clinical history revealed a consistent macrocephaly and mild retardation, no signs of acute neurological deterioration. MRI shows symmetrical fronto-parieto-occipital subdural collections (*asterisk*), atrophy of the frontal lobes, and bilateral wide Sylvian fissures (*arrows*). The imaging findings are consistent with GA1. The work-up for child abuse revealed no concern

The clinically diagnosed group had a median age of 17.9 years (range 10–33 years) at the time of this study. The head circumference defined in standard deviations (SD) was on average +1.04 SD (range −2 SD to >3 SD). Neuroimaging studies were available in 15 patients of whom 12 had one or more MRI scans (range 1–3), 6 had one or more CT scans (range 1–3), and 3 patients had undergone both MRI and CT. In this latter group, 1 child (Table 1, case 14) was diagnosed with a SDH (Fig. 1).

## Newborn screening group

None of the patients in the NBS group had family members previously diagnosed with GA1. The median age of these patients at time of this study was 4.0 years (range 11 months–7 years). With a median head circumference of +0.69 SD (range −1 SD to +2.5 SD), the NBS patients had a similar deviation to the patients in the clinical diagnosis group. Five patients did not have CT or MRI scans, 2 patients had one or more MRI scans (range 1–2) and 1 had a CT scan once. There were no cases of SDH in this group.

## Discussion

Our study demonstrated a rather low incidence (5.9 %) of SDH, in GA1 patients identified before the use of NBS, compared to the reported incidence of SDH which ranges from 10 to 30 % in children with GA1 in most literature reports [4, 8]. There is however one large retrospective study with a SDH incidence of 0 % on MRI [5]. The lower overall birth incidence in the Dutch population compared to previously internationally reported incidences needs to be taken into account. Because of a national-based database, we are quite confident we did not miss any cases. This low incidence might be due to prenatal counseling. Furthermore, none of the 3 patients who were diagnosed with GA1 through NBS and of whom imaging results were available had a SDH. The overall incidence in our study population is 4.0 %. The aim of early diagnosis by NBS is to start treatment as early as possible. Even though up to 25 % of untreated patients remained completely asymptomatic, the aim of the treatment is to reduce the risk of CNS complications, including the risk of SDH development [13]. The only patient in our cohort in whom SDH was diagnosed was born before the introduction of GA1 in NBS in the Netherlands. SDHs are thus less frequently seen in GA1 children than has been reported by some. Nevertheless, these children are still at risk of significant hemorrhage, even if diagnosed by NBS and treated according to recent guideline recommendations.

Although all Dutch GA1 patients were included, the relative small amount of patients remains a limitation. Another limitation of this study is that 2 out of 17 of the clinical patients and 5 out of 8 of the NBS group patients did not have any neuroimaging results. However, these patients did not have any clinical signs of a SDH, such as a change in head circumference, an altered level of consciousness, vomiting, paralysis, seizures, or other relevant neurological symptoms, during their out-patient clinic follow-up, thus obviating the need of neuroimaging. We therefore feel confident that no cases of SDH were missed.

The most common cause of SDHs is AHT [11], other causes include birth trauma, accidental trauma, meningitis, coagulation disorders, and metabolic disorders including GA1 [7]. In the past, SDH cases due to GA1 have been misdiagnosed as AHT [12]. GA1, although a rare cause of SDHs, is generally included in the forensic-medical differential diagnosis of SDH due to suspected AHT [13], which has serious social and medico-legal consequences [6]. Unnecessary delay in AHT diagnosis, for instance by GA1 testing, is thus avoidable. The outcome of AHT with SDHs is poor, with a mortality of 15–23 % [3, 7, 11, 21]. Surviving children have a high morbidity rate of 64 % of which half is severe [3]. Abuse should thus at least be considered and investigated. It is important to keep in mind that a diagnosis of GA1 does not rule out the possibility of AHT, since both conditions can coexist [8, 26]. In addition, disabled and chronically ill children are even more prone to become victims of abuse [8, 10].

This study shows an overall low incidence of SDHs in patients with glutaric aciduria type 1 in our Dutch population. The incidence might become even lower in the future due to early treatment after positive NBS. This combined with the high NBS compliance of 99.8 % and the fact that SDHs in GA1 appear to occur usually in the presence of characteristic brain abnormalities on imaging, we recommend that GA1 should not routinely be a part of the differential diagnosis of children with unexplained SDHs in the absence of imaging characteristics suggestive of GA1.

## Abbreviations

CBS: Central Bureau of Statistics
CNS: Central nervous system
CSF: Cerebrospinal fluid
GA1: Glutaric aciduria type 1
GCDH: Glutaryl-CoA dehydrogenase
NBS: Newborn screening
SD: Standard deviations
SDH: Subdural hematoma
